# Blockade of tumor cell-intrinsic PD-L1 signaling enhances AURKA-targeted therapy in triple negative breast cancer

**DOI:** 10.3389/fonc.2024.1384277

**Published:** 2024-05-30

**Authors:** Andrew Takchi, Minzhi Zhang, Mohammad Jalalirad, Roberto Leon Ferre, Royal Shrestha, Tufia Haddad, Jann Sarkaria, Ann Tuma, Jodi Carter, Hillman David, Karthik Giridhar, Liewei Wang, Carol Lange, Urban Lendahl, James Ingle, Matthew Goetz, Antonino Bonaventura D’Assoro

**Affiliations:** ^1^ Department of Oncology, Mayo Clinic College of Medicine, Rochester, MN, United States; ^2^ Department of Pathology, Mayo Clinic College of Medicine, Rochester, MN, United States; ^3^ Department of Pharmacology, University of Minnesota, Minneapolis, MN, United States; ^4^ Department of Cell and Molecular Biology, Karolinska Institutet, Stockholm, Sweden

**Keywords:** triple negative breast cancer, immunotherapy, cancer cell plasticity, small molecule inhibitor, organ metastases

## Abstract

Triple negative breast cancer (TNBC) accounts for 15–20% of all breast cancers and mainly affects pre-menopausal and minority women. Because of the lack of ER, PR or HER2 expression in TNBC, there are limited options for tailored therapies. While TNBCs respond initially to standard of care chemotherapy, tumor recurrence commonly occurs within 1 to 3 years post-chemotherapy and is associated with early organ metastasis and a high incidence of mortality. One of the major mechanisms responsible for drug resistance and emergence of organ metastasis is activation of epithelial to mesenchymal transition (EMT) reprogramming. EMT-mediated cancer cell plasticity also promotes the enrichment of cancer cells with a CD44^high^/CD24^low^ and/or ALDH^high^ cancer stem-like phenotype [cancer stem cells (CSCs)], characterized by an increased capacity for tumor self-renewal, intrinsic drug resistance, immune evasion and metastasis. In this study we demonstrate for the first time a positive feedback loop between AURKA and intra-tumoral PD-L1 oncogenic pathways in TNBC. Genetic targeting of intra-tumoral PD-L1 expression impairs the enrichment of ALDH^high^ CSCs and enhances the therapeutic efficacy of AURKA-targeted therapy. Moreover, dual AURKA and PD-L1 pharmacological blockade resulted in the strongest inhibition of tumor growth and organ metastatic burden. Taken together, our findings provide a compelling preclinical rationale for the development of novel combinatorial therapeutic strategies aimed to inhibit cancer cell plasticity, immune evasion capacity and organ metastasis in patients with advanced TNBC.

## Introduction

Triple negative breast cancer (TNBC) accounts for 15–20% of all breast cancers and mainly affects pre-menopausal and minority women (1). Because of the lack of ER, PR or HER2 expression in TNBC, there are until now limited options for tailored therapies (2). TNBC is therefore treated with standard of care chemotherapy, using genotoxic stress-inducing drugs such as cisplatin and carboplatin, or microtubule-stabilizing agents such as taxanes (paclitaxel or docetaxel). While TNBCs respond initially to chemotherapy, tumor recurrence commonly occurs within 1 to 3 years post-chemotherapy and is associated with early emergence of organ metastasis and a high incidence of mortality (3).

Cancer cell plasticity represents one of the major hindrances in eradicating organ metastasis because it promotes high self-renewal capacity, intrinsic resistance to chemotherapeutic agents and immune evasion ability to cancer cells (4–6). One of the central mechanisms responsible for the development of cancer cell plasticity is epithelial to mesenchymal transition (EMT) reprogramming. Activation of EMT reprogramming drives the transition from a polarized epithelial phenotype to an elongated fibroblastoid-like phenotype that typifies the morphology of aggressive breast tumors (7). Importantly, EMT-induced cancer cell plasticity is also linked to high immune evasion ability of cancer cells (8).

Activation of EMT reprogramming also induces the enrichment of cancer cells with a CD44^high^/CD24^low^ and/or ALDH^high^ cancer stem-like phenotype characterized by an increased capacity for tumor self-renewal, intrinsic drug resistance and high metastatic proclivity (9). Elevated levels and activity of ALDH (aldehyde dehydrogenase) has been detected in normal stem cells and cancer stem-like cells (CSCs) (10). The molecular mechanisms by which high ALDH activity induces intrinsic drug resistance is primarily through ALDH-mediated detoxification of toxic aldehyde intermediates produced in cancer cells following treatment with chemotherapy (11).

Several oncogenic signaling pathways induce EMT-mediated cancer cell plasticity and tumor stemness. Aurora-A kinase (AURKA) is a serine/threonine oncoprotein kinase that localizes to centrosomes and mitotic spindles of dividing cells (12). AURKA controls centrosome duplication and spindle formation for appropriate chromosome segregation during mitosis safeguarding the maintenance of chromosomal stability (13). Aberrant AURKA activity plays a major role in tumor progression through development of centrosome amplification and chromosomal instability (CIN) (14, 15). When AURKA is overexpressed in human breast tumors, it is commonly associated with a *basal-like* phenotype and poor prognosis (16). Significantly, deregulated AURKA activity also induces EMT-mediated cancer cell plasticity, tumor stemness and drug resistance in breast cancer (17). Because of its role in tumor progression and poor clinical outcomes, AURKA is a promising druggable target in cancer and small molecule inhibitors of AURKA activity are under preclinical and clinical investigation (18, 19). The AURKA inhibitor alisertib has shown promising therapeutic efficacy in metastatic ER+ breast cancer patients that were resistant to endocrine therapy and CDK4/6 inhibitors (20). Nonetheless, AURKA inhibitors as monotherapy did not show high therapeutic efficacy in TNBC, and the combination with standard of care chemotherapy may limit their clinical development due to overlapping toxicities (21). The limited activity of AURKA inhibitors in TNBC could be linked to high cancer cell plasticity and activation of alternative oncogenic pathways that will sustain cancer stemness and promote early tumor relapse and progression (22, 23).

Recent findings have shown that CSCs also display high intra-tumoral PD-L1 expression that plays a critical role in inducing tumor immune escape through CD8+ T-cells exhaustion (24). Immune checkpoint inhibitors (ICIs) are emerging targeted therapeutic drugs for many cancers and are based on blocking the interaction between PD-L1 and PD-1 receptors. High expression of the PD-L1 ligand is associated with poor prognosis in breast cancer (25) and ICIs have been FDA-approved as a neoadjuvant or adjuvant therapy for TNBC.

In this study we demonstrate for the first time a positive feedback loop between AURKA and intra-tumoral PD-L1 oncogenic pathways in TNBC. Remarkably, genetic targeting of intra-tumoral PD-L1 expression impairs the enrichment of ALDH^high^ CSCs and enhances the therapeutic efficacy of AURKA-targeted therapy. Moreover, dual AURKA and PD-L1 pharmacological blockade resulted in the strongest inhibition of tumor growth and organ metastatic burden. The efficacy of dual AURKA and PD-L1 pharmacological blockade was corroborated in a unique model of durvalumab-resistant TNBC cells. Taken together, our findings provide the strong preclinical rationale for the development of novel combinatorial therapeutic strategies aimed at inhibiting cancer cell plasticity, immune evasion capacity and organ metastasis in patients with advanced TNBC.

## Materials and methods

**Mayo Clinic cohort:** Breast tumor biospecimens were obtained from the Mayo Clinic Cancer Center. Median expression of low and high AURKA was evaluated by Immunohistochemistry (IHC) and intensity of AURKA staining was reported using the Aperio Whole Cell Quant Application package and ImageScope viewing software. AURKA rabbit monoclonal antibody (#91590) from Cell Signaling was employed in these studies.

**Established breast cancer cell lines:** The human breast cancer cell lines MDA-MB 231 and BT-474 were obtained from ATCC (Manassas, VA, USA). SUM149-PT cancer cells were kindly provided by Dr. Couch’s laboratory (Mayo Clinic, Rochester, MN, USA). All cell lines were maintained in DMEM medium containing 5mM glutamine, 1% penicillin/streptomycin and 10% FBS at 37 C in 5% CO2 atmosphere. All cell lines were tested for mycoplasma contamination.

**Patient-derived TNBC cells:** TNBC-M40 cells were isolated from patient-derived brain metastasis TNBC xenograft models that were generated at the Mayo Clinic Cancer Center (19). To establish cultured TNBC-M40 cells, patient-derived xenograft metastatic models were excised from killed animals, minced using sterile scissors, transferred to complete culture medium and fibroblast-free TNBC-M40 cells were cultured in low-adherent flasks supplemented with MammoCult™ medium (STEMCELL 05620) and propagated in culture as 3D-Organoids and used for this study.

**Immunoblot and immunofluorescence assays:** Antibodies employed to perform these studies were the followings: AURKA (AbCam 13824, Cambridge, MA, USA); vimentin (AbCam 52903, Cambridge, MA, USA); PD-L1, CD44, CD-8, Cleaved-PARP, α-tubulin (Sigma T9026, St. Louis, Missouri, USA) and GAPDH (AbCam 9485, Cambridge, MA, USA). FITC and Rodhamine secondary antibodies were obtained from Molecular Probes (Eugene, OR, USA).

**ALDH activity assay:** ALDH activity was detected by FACS analysis using the AldeRed ALDH Detection Assay kit (Millipore Sigma SCR150, USA) according to the manufacturer’s instructions.

**Tumor xenografts:** Procedures established by the Institutional Animal Care and Use Committee based on US NIH guidelines for the care and use of laboratory animals were followed for all experiments (IACUC: A00002634–17-R20). Establishment of MDA-MB 231 LM xenografts: 4 weeks old non-ovariectomized female NSG mice and humanized-CD34+ female NSG mice (the Jackson Laboratory) were anesthetized by exposure to 3% isoflurane and injected into the mammary fat pad with 1× 106 cells (infected with a luciferase lenti-vector to detect the presence of distant metastasis) suspended in 50 ul of 50% Matrigel (BD Bioscience, Bedford, MA, USA). After 2 weeks tumor growth, mice were randomized and treated with alisertib (oral gavage) and/or atezolizumab 3 times/week for 3 weeks. After drug treatment, tumor relapse was monitored for additional 3 weeks or when the tumor xenografts reached a volume comparable to control groups. Tumor volume was measured 3 times/week using a digitized caliper. Following drug treatment, mice were sacrificed, and organ metastatic burden was determined *ex-vivo* using the Xenogen imaging system.

**Scientific rigor and statistical analysis:** FACS, immunoblot and Immunofluorescence assays were run in triplicate or for 3 independent runs (*+/- S.D.*). The average read-out of the triplicates from each run was determined and a 95% t-confidence interval for the difference between was constructed using IBM SPSS Statistics. Different triple-negative breast cancer cell lines and patient-derived xenografts (PDXs) were employed in this study to increase the power to detect the effect size. The nonparametric Mann-Whitney t test (Statview software) was used to determine the significance of the relative tumor volumes for treated versus untreated TNBC xenograft groups. Using an initial sample size of 5 animals per group, a two-sided (alpha=0.05), two sample t-test for assessing whether the difference in mean tumor burden differs significantly between a particular pair of treatment groups will have a power of 90% to detect a difference of 1.6 standard deviation (*SD*). For each xenograft (treated and control groups), the difference in the percentage of organ metastatic burden was assessed. Animals were examined every day and body weight, and primary tumor size was measured 3 times per week. The statistical difference in tumor growth between control and treated groups with alisertib and/or atezolizumab were compared among each other for their evolution and possible responses to drug treatment using the cutting-edge *R-package TumGrowth* software tool allowing to carry out a series of statistical comparisons across or between groups of tumor growth curves (https://kroemerlab.shinyapps.io/TumGrowth/) (26).

## Results and discussion

**Figure 1:**
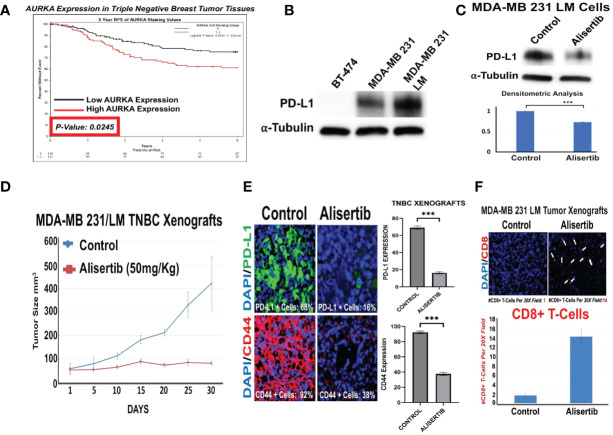
AURKA Induces Intra-Tumoral PD-L1 Expression in Triple Negative Breast Cancer Cells: **(A)** Immunohistochemistry analysis (IHC) was performed on a selected cohort of 269 tumor tissues from TNBC patients. AURKA protein was stained with a mouse monoclonal antibody (Cell Signaling). High and low AURKA median expression was associate with reduced RFS during a 5 year follow-up period. **(B)** Immunoblot analysis showing intra-tumoral PD-L1 expression in BT-474 (negative control), MDA-MB 231 and variant MDA-MB 231 LM cells. **(C)** Immunoblot analysis showing intra-tumoral PD-L1 expression in MDA-MB 231 LM cells before and after treatment with 50 nM Alisertib. Densitometric analysis showing the fold change of PD-L1 protein levels normalized to alpha-Tubulin was performed using ImageJ-NIH Software. **(D)** 1x10^6^ MDA-MB 231 LM cells were transplanted into the right 4^th^ mammary fat pad of 20 female humanized-CD34+ NSG mice. After 2 weeks of tumor growth, animals were randomized into two groups (5 animal/group) and treated with saline solution placebo (control group) or alisertib (50 mg/Kg) oral gavage, 3 times per week for 30 days. Tumor growth was measured using digital calipers. **(E)** Immunofluorescence analysis showing representative images of PD-L1 and CD44 expression in MDA-MB 231/LM tumor xenografts (5 animal/group) from control and alisertib-treated groups. PD-L1 was labeled in green with FITC and CD44 was labeled in red with rodhamine. Nuclei were labeled in blue with DAPI. The percentage of PD-L1+ cells represents the average of three independent experiments. **(F)** Immunofluorescence analysis showing representative images of CD8+ T-cells in MDA-MB 231 LM tumor xenografts (5 animal/group) from control and alisertib-treated groups. CD8+ T-cells were labeled in Red with rodhamine. Nuclei were labeled in blue with DAPI. The number of CD8+ T-cells per 20X field represents the average of three independent experiments. ***, <0.001.

In order to define the association between increased AURKA protein expression and shorter recurrence free survival (RFS) of TNBC patients, we analyzed AURKA protein expression by immunohistochemistry in a unique cohort of tumor tissues established at the Mayo Clinic Cancer Center from 269 TNBC patients. 136 patients showed low AURKA expression, while 133 patients showed high AURKA expression. High AURKA expression was significantly associated with reduced RFS during a five-year follow up period (**Figure 1A**). Because AURKA and intra-tumoral PD-L1 oncogenic signaling pathways induce EMT and cancer cell plasticity, we investigated the extent to which AURKA kinase activity may regulate intra-tumoral PD-L1 expression in TNBC cells. We have established unique MDA-MB 231/LM cells (isolated from lung metastasis) (19) that show high endogenous levels of PD-L1 (**Figure 1B**). MDA-MB 231/LM cells were treated with alisertib, and PD-L1 expression was assessed using western blot analysis. Alisertib reduced intra-tumoral PD-L1 expression (**Figure 1C**). To define *in vivo* the effect of AURKA pharmacological blockade on tumor growth and inhibition of immune evasion capacity, we established MDA-MB 231/LM xenografts in humanized NSG-CD34+ female mice. Animals were treated with placebo or alisertib (50mg/Kg oral gavage three times per week). Alisertib-treated tumor xenografts showed a significant reduction of tumor growth that was linked to down-regulation of PD-L1 and CD44 expression, and an increase of CD8+ T-cells infiltration compared to control group (**Figures 1D–F**). Taken together, these results indicate that pharmacological blockade of AURKA activity impairs tumor stemness and increases CD8+ T-cells infiltration through reduction of intra-tumoral PD-L1 expression.

**Figure 2:**
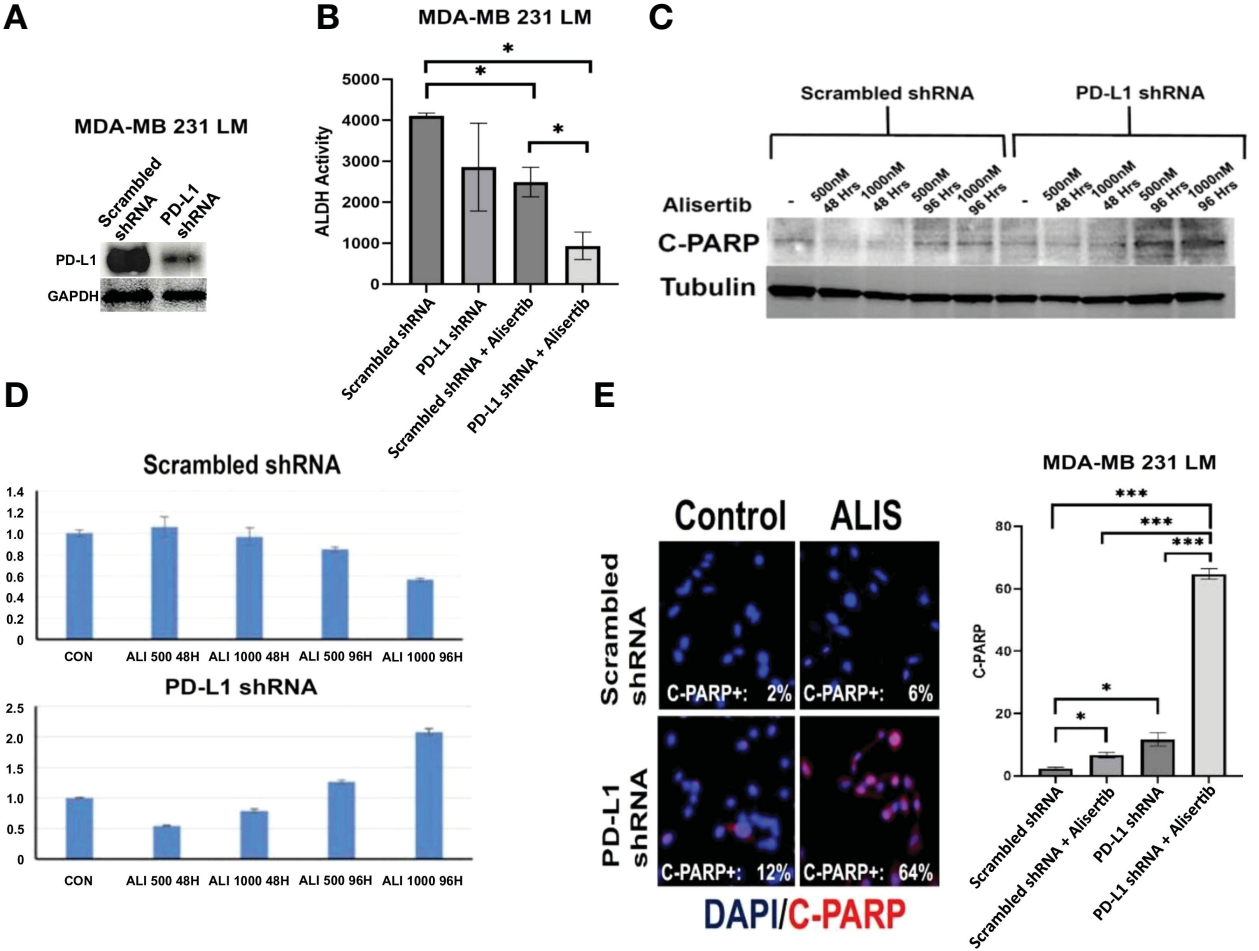
Genetic Targeting of Intra-Tumoral PD-L1 Impairs Tumor Stemness Capacity: **(A)** Immunoblot analysis showing intra-tumoral PD-L1 expression in MDA-MB 231 LM cells infected with scrambled (control) or PD-L1 lenti-shRNAs. **(B)** MDA-MB 231 LM cells were infected with scrambled (control) or PD-L1 lenti-shRNAs and treated with alisertib. After 48 hours incubation, ALDH activity was detected with Aldefluor kit and measured by FACS analysis on 10,000 events. ALDH inhibitor DEAB was used as control for each sample. Graph showing experiments performed in duplicate (+/- S.E.M. and P value < 0.05). **(C)** C-PARP expression was assessed by immunoblot assay after 48 and 96 hours of treatment with alisertib. **(D)** Densitometric analysis showing the fold change of C-PARP expression levels normalized to alpha-Tubulin was performed using ImageJ-NIH Software. **(E)** Immunofluorescence analysis showing representative images of C-PARP positive MDA-MB 231 LM cells from control and alisertib (ALIS)-treated cells. C-PARP positive MDA-MB 231 LM cells were labeled in Red with rodhamine. Nuclei were labeled in blue with DAPI. Graph showing the percentage of C-PARP positive MDA-MB 231 LM cells from three independent experiments (+/- S.D. and P value < 0.05). *, <0.05; ***, <0.001.

To define the extent to which intra-tumoral PD-L1 expression was necessary to induce the enrichment of ALDH^high^ CSCs, restraining the therapeutic efficacy of AURKA-targeted therapy, *ex-vivo* MDA-MB 231/LM cells were infected with either scrambled shRNAs or PD-L1 shRNAs lenti-vectors (**Figure 2A**) and treated with alisertib for 48 hours. Remarkably, PD-L1 genetic targeting enhanced the efficacy of alisertib in reducing the enrichment of ALDH^High^ CSCs (**Figure 2B** and **Supplementary Figure 1**). Because ALDH activity is necessary to induce self-renewal capacity of cancer cells and resistance to anticancer drugs-induced apoptosis (19), MDA-MB 231/LM cells infected with either scrambled shRNAs or PD-L1 shRNAs lenti-vectors were treated with alisertib for 48 hours and cleaved-PARP expression and cellular localization was assessed as a biomarker of apoptosis. PD-L1 genetic targeting enhanced alisertib-induced apoptosis of cancer cells (**Figures 2C–E**). These findings strongly demonstrate that tumor intrinsic PD-L1 signaling has immune independent oncogenic functions that sustain the enrichment of ALDH^high^ CSCs and induce resistance to AURKA-targeted therapy in TNBC cells.

**Figure 3:**
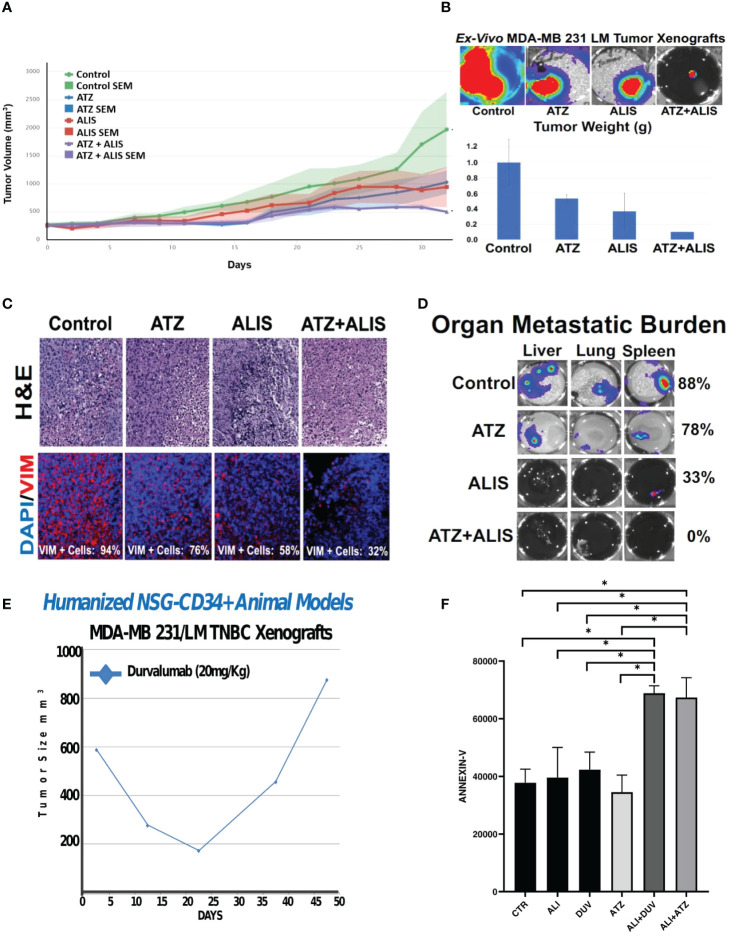
Dual AURKA and PD-L1 Pharmacological Blockade Inhibits Tumor Growth and Metastasis: **(A)** 1x10^6^ MDA-MB 231 LM cells infected with luciferase lenti-vectors were transplanted into the right 4th mammary fat pad of 20 female NSG mice. After 2 weeks of tumor growth, animals were randomized into four groups (5 animal/group) and treated with saline solution placebo (control group), alisertib (ALIS) (25 mg/Kg) oral gavage, atezolizumab (ATZ) (20mg/Kg) I.P. injections and combination 3 times per week for 33 days. The statistical difference in tumor growth between control and treated groups were compared among each other for their evolution and possible responses to drug treatment using the *R-package TumGrowth* software. **(B)** After 33 days, animals were sacrificed and luciferase intensity was assessed *ex-vivo* in primary tumor xenografts using the Xenogen instrument. Tumor xenografts were weighted to corroborate *in vivo* tumor growth inhibition after AURKA and PD-L1 pharmacological co-targeting. There was also a significant reduction between control groups and animals treated with alisertib + atezolizumab. **(C)** H&E of tumor xenograft tissues after *in vivo* treatment with alisertib and/or atezolizumab. Immunofluorescence analysis showing representative images of vimentin expression in tumor xenograft tissues after *in vivo* treatment with alisertib and/or atezolizumab. Vimentin was labeled in Red with Rodhamine. Nuclei were labeled in blue with DAPI. **(D)** *Ex-Vivo* organs isolated from animals after *in vivo* treatment with alisertib and/or atezolizumab. Organ metastatic burden was measured in liver, lung and spleen tissues using the Xenogen instrument. **(E)** 1x10^6^ MDA-MB 231 LM cells were transplanted into the right 4th mammary fat pad of Humanized NSG-CD34+ female mice (five) and treated with durvalumab (20mg/Kg) three times/week for 50 days. **(F)** *Ex-vivo* MDA-MB 231 LM durvalumab-resistant cells were treated with 100 ng atezolizumab, 100 ng durvalumab and/or 50 nM Alisertib for four days. Real-Time assay (*IncuCyte*) was employed to quantify apoptotic cells (Annexin-V) before and after drug treatment. Three independent experiments were performed (+/- S.E.M. and P value < 0.05). *, <0.05.

Because AURKA and PD-L1 are both *druggable* oncogenic signaling pathways, we aimed to define *in vivo* the therapeutic efficacy of dual AURKA and PD-L1 pharmacological blockade. MDA-MB 231/LM tumor xenografts were established in immunocompromised female NSG mice. Animals were treated with placebo (control group), atezolizumab (anti-PD-L1, 20 mg/Kg I.P. injections) and/or alisertib (25mg/Kg oral gavage) three times per week (**Figure 3A**). The combination of atezolizumab and alisertib was well-tolerated because animals did not experience weight loss >15% (data not shown). Combination of atezolizumab and alisertib showed the strongest tumor growth inhibition (**Figures 3A, B**). Noteworthy, combination of atezolizumab and alisertib triggered the reduced expression of the mesenchymal marker vimentin in tumor xenografts that was linked to lack of organ metastasis compared to atezolizumab or alisertib as monotherapy (**Figures 3C, D**). Because MDA-MB 231/LM tumor xenografts were established in immunocompromised NSG mice, the therapeutic efficacy of PD-L1 pharmacological blockade in enhancing alisertib efficiency was not immune-driven but it was exclusively linked to inhibition of tumor-intrinsic PD-L1 oncogenic signaling pathway. To substantiate the therapeutic efficacy of dual AURKA and PD-L1 pharmacological targeting, we employed a unique MDA-MB 231/LM xenograft model that developed *in vivo* resistance to durvalumab (**Figure 3E**), an anti-PD-L1 humanized antibody that improves long-term outcome in TNBC patients (27). Remarkable, combination of alisertib with FDA-approved ICIs (atezolizumab or durvalumab) resulted in the highest induction of apoptosis in *ex-vivo* MDA-MB 231/LM durvalumab-resistant cells (**Figure 3F**).

**Figure 4:**
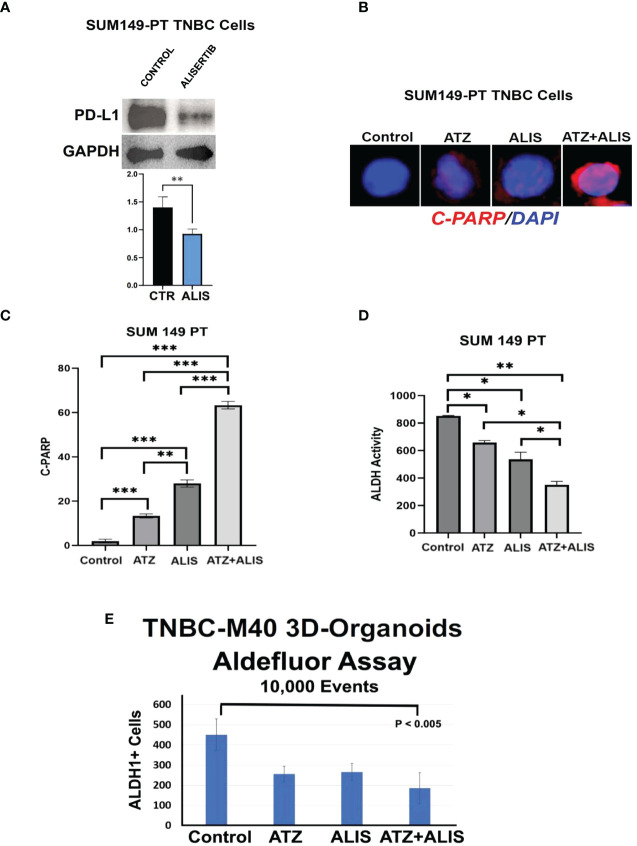
Dual AURKA and PD-L1 Pharmacological Blockade Induces Apoptosis and Inhibits ALDH Activity: **(A)** Immunoblot analysis showing intra-tumoral PD-L1 expression in SUM149-PT cells before and after treatment with 50 nM Alisertib. Densitometric analysis showing the fold change of PD-L1 protein levels normalized to GAPDH was performed using ImageJ-NIH Software. **(B)** Immunofluorescence analysis showing representative images of C-PARP positive SUM149-PT cells from control, alisertib and/or atezolizumab-treated cells for 48 hours. C-PARP positive SUM149-PT cells were labeled in Red with Rodhamine. Nuclei were labeled in blue with DAPI. **(C)** Graph showing the percentage of C-PARP positive SUM149-PT cells from three independent experiments (+/- S.D. and P value < 0.05). **(D)** ALDH activity was assessed in SUM149-PT cells with Aldefluor kit and measured by FACS analysis on 10,000 events. ALDH inhibitor DEAB was used as control for each sample. Graph showing experiments performed in duplicate (+/- S.E.M. and P value < 0.05). **(E)** 3D-Organoids were treated with 100 ng Atezolizumab and/or 50 nM Alisertib. After 48 hours incubation, ALDH activity was detected with Aldefluor kit and measured by FACS analysis on 10,000 events. ALDH inhibitor DEAB was used as control for each sample. Graph showing three independent experiments performed in triplicate (+/- S.D. and P value < 0.05). *, <0.05; **, <0.01; ***, <0.001.

To corroborate the immune independent effects of dual AURKA and PD-L1 pharmacological blockade, we also assessed the combination of alisertib and atezolizumab in SUM-PT 149 TNBC cells (19). Treatment of SUM-PT 149 cells with alisertib induced PD-L1 down-regulation (**Figure 4A**), reinforcing the pivotal role of AURKA kinase activity in inducing intra-tumoral PD-L1 expression in TNBC cells. Combination of alisertib and atezolizumab resulted in the strongest induction of apoptosis compared to alisertib or atezolizumab as monotherapy (**Figures 4B, C**). Importantly, dual AURKA and PD-L1 pharmacological blockade also induced the strongest inhibition of ALDH activity compared to alisertib or atezolizumab as monotherapy (**Figure 4D** and **Supplementary Figure 2**). To investigate the therapeutic efficacy of AURKA and PD-L1 pharmacological co-targeting in clinically relevant models, we established 3D-Organoids from an exclusive metastatic Patient Derived Xenograft (PDX), TNBC-M40 (19). 3D-Organoids were treated with atezolizumab and/or Alisertib for 48 hours and ALDH activity was measured by Aldefluor assay. Combination of atezolizumab and alisertib induced the strongest reduction of ALDH^high^ CSCs compared to atezolizumab or alisertib as single agents (**Figure 4E**).

## Conclusion

Because FDA-approved targeted therapies are currently limited for metastatic TNBC, there is an urgent need for the discovery of effective novel therapeutic strategies aimed to significantly improve the overall survival of patients with advanced TNBC. This study provides a strong preclinical rationale that dual AURKA and PD-L1 pharmacological blockade is an effective combinatorial therapeutic approach to inhibit TNBC progression through selective eradication of ALDH^high^ CSCs that are responsible for the high cancer cell plasticity and immunosuppressive activity. Pharmacological blockade of AURKA/PD-L1 oncogenic axis represents a major breakthrough in the treatment of metastatic TNBC, because it will lead to the development of novel combinatorial targeted therapies expected to meaningfully increase the progression-free and overall survival of patients with metastatic TNBC that are refractory to standard of care chemotherapy and show limited response to FDA-approved ICIs.

## Data availability statement

The raw data supporting the conclusions of this article will be made available by the authors, without undue reservation.

## Ethics statement

Ethical approval was not required for the studies on humans in accordance with the local legislation and institutional requirements because only commercially available established cell lines were used. The animal study was approved by mayo clinic institutional animal committee. The study was conducted in accordance with the local legislation and institutional requirements.

## Author contributions

ATa: Conceptualization, Data curation, Formal analysis, Investigation, Methodology, Writing – original draft, Project administration, Software, Supervision, Validation, Visualization, Writing – review & editing. MZ: Formal analysis, Methodology, Software, Validation, Writing – review & editing. MJ: Formal analysis, Investigation, Methodology, Writing – review & editing. RF: Data curation, Investigation, Methodology, Writing – review & editing. RS: Formal analysis, Methodology, Writing – review & editing. TH: Investigation, Methodology, Writing – review & editing. JS: Formal analysis, Investigation, Methodology, Writing – review & editing. ATu: Investigation, Methodology, Resources, Writing – review & editing. JC: Formal analysis, Investigation, Methodology, Resources, Writing – review & editing. HD: Data curation, Methodology, Supervision, Writing – review & editing. KG: Formal analysis, Investigation, Methodology, Writing – review & editing. LW: Formal analysis, Investigation, Methodology, Resources, Writing – review & editing. CL: Investigation, Methodology, Writing – review & editing. UL: Formal analysis, Investigation, Methodology, Writing – review & editing. JI: Formal analysis, Investigation, Methodology, Writing – review & editing. MG: Formal analysis, Investigation, Methodology, Writing – review & editing. AD’: Conceptualization, Data curation, Formal analysis, Investigation, Methodology, Project administration, Resources, Supervision, Visualization, Writing – original draft, Writing – review & editing.

## Supplementary material

Supplementary Figure 1 MDA-MB 231 LM cells were infected with scrambled (control) or PD-L1 lenti-shRNAs and treated with alisertib. After 48 hours incubation, ALDH activity was detected with Aldefluor kit and measured by FACS analysis on 10,000 events. ALDH inhibitor DEAB was used as control for each sample. Experiments were performed in duplicate.

Supplementary Figure 2 ALDH activity was assessed in SUM149-PT cells with Aldefluor kit and measured by FACS analysis on 10,000 events. ALDH inhibitor DEAB was used as control for each sample. Experiments were performed in duplicate.
