# Supplementary figures and images for: Blockade of tumor cell-intrinsic PD-L1 signaling enhances AURKA-targeted therapy in triple negative breast cancer
Source: Front Oncol. 2024 May 30;14:1384277. doi: 10.3389/fonc.2024.1384277 (PMC11169658; doi:10.3389/fonc.2024.1384277)

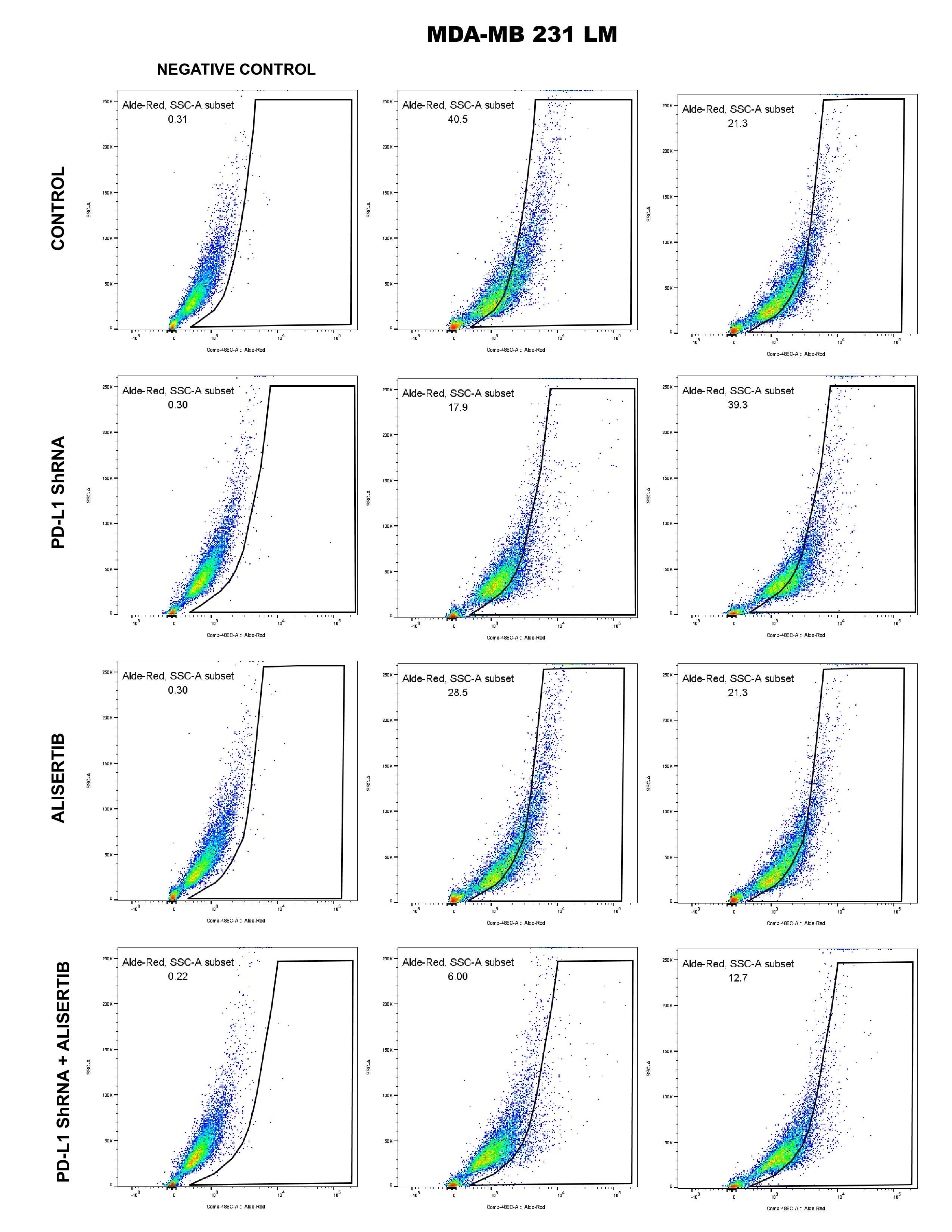

Supplement: Supplementary Figure 1 — MDA-MB 231 LM cells were infected with scrambled (control) or PD-L1 lenti-shRNAs and treated with alisertib. After 48 hours incubation, ALDH activity was detected with Aldefluor kit and measured by FACS analysis on 10,000 events. ALDH inhibitor DEAB was used as control for each sample. Experiments were performed in duplicate. [file Image_1.jpeg]

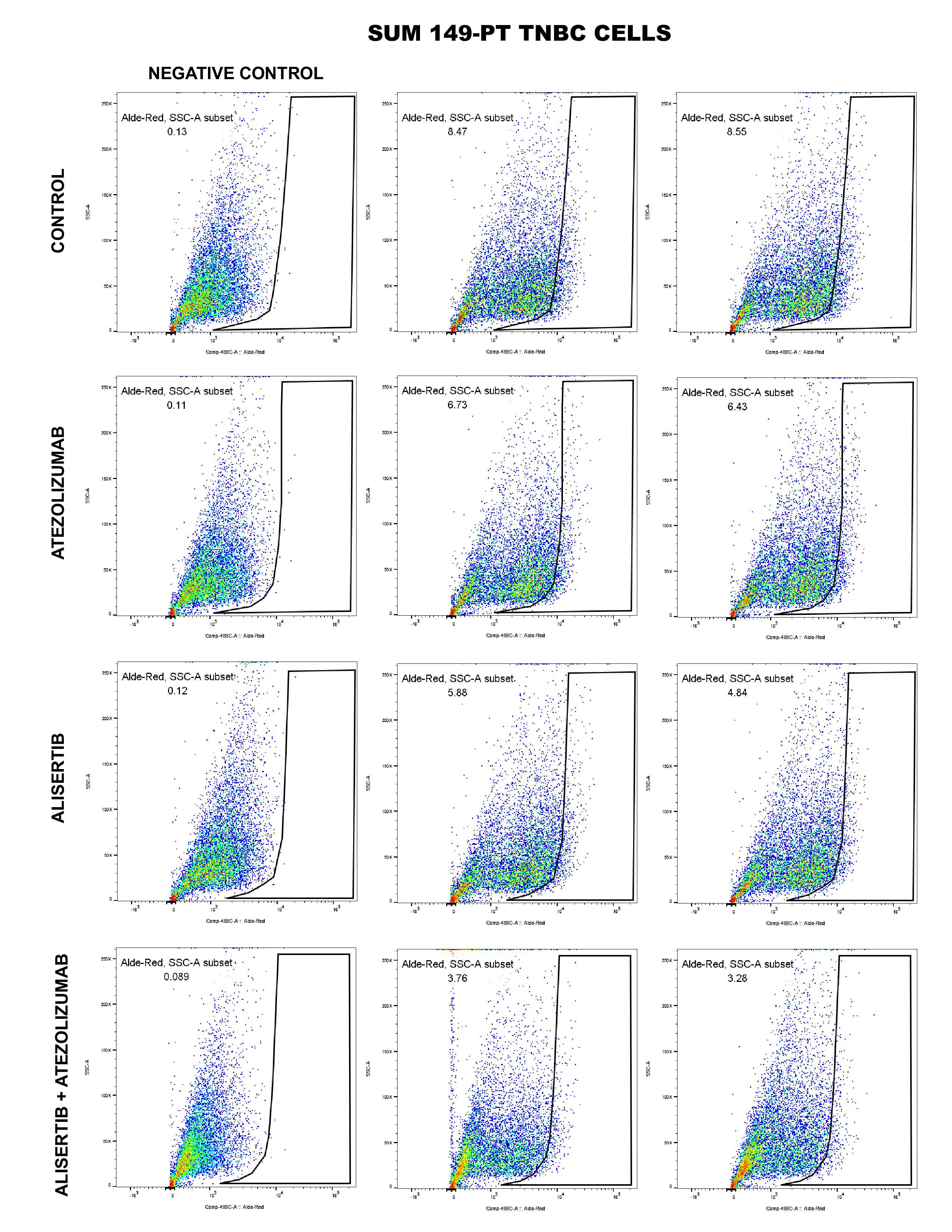

Supplement: Supplementary Figure 2 — ALDH activity was assessed in SUM149-PT cells with Aldefluor kit and measured by FACS analysis on 10,000 events. ALDH inhibitor DEAB was used as control for each sample. Experiments were performed in duplicate. [file Image_2.jpeg]
